# Anaemia and iron deficiency in heart failure: epidemiological gaps, diagnostic challenges and therapeutic barriers in sub-Saharan Africa

**DOI:** 10.5830/CVJA-2017-001

**Published:** 2017

**Authors:** Abel Makubi, Johnson Lwakatare, Julie Makani, Abel Makubi, Lars Rydén, Lars H Lund,, Johnson Lwakatare, Julie Makani, Okechukwu S Ogah, Lars H Lund, Julie Makani

**Affiliations:** School of Medicine, Muhimbili University of Health and Allied Sciences, Dar es Salaam, Tanzania; School of Medicine, Muhimbili University of Health and Allied Sciences, Dar es Salaam, Tanzania; School of Medicine, Muhimbili University of Health and Allied Sciences, Dar es Salaam, Tanzania; Cardiology Unit, Department of Medicine K2, Karolinska Institutet, Stockholm, Sweden; Cardiology Unit, Department of Medicine K2, Karolinska Institutet, Stockholm, Sweden; Cardiology Unit, Department of Medicine K2, Karolinska Institutet, Stockholm, Sweden; Cardiology Unit, Department of Medicine K2, Karolinska Institutet, Stockholm, Sweden; Cardiology Unit, Department of Medicine K2, Karolinska Institutet, Stockholm, Sweden; Division of Cardiology, Department of Medicine, University College Hospital, Ibadan, Nigeria; Department of Cardiology, Karolinska University Hospital, Stockholm, Sweden; Nuffield Department of Clinical Medicine, University of Oxford, London, United Kingdom

**Keywords:** heart failure, anaemia, iron deficiency, review, epidemiology, therapy, sub-Saharan Africa

## Abstract

Anaemia and iron deficiency (ID) are common and of prognostic importance in heart failure (HF). In both conditions the epidemiology, diagnosis and therapies have been extensively studied in high-income countries but are still largely unexplored in sub-Saharan Africa (SSA).

The lack of adequate and robust epidemiological data in SSA makes it difficult to recognise the significance of anaemia and ID in HF. From a clinical perspective, less attention is paid by clinicians to screening for anaemia in HF, and as far as interventions are concerned, there are no clinical trials in SSA that provide guidance on the appropriate interventional approach. Therefore studies are needed to provide more insight into the burden and peculiarities of and intervention for anaemia and ID in HF in SSA, where the pathophysiology might be different from that in high-income countries.

There is increasing appreciation that targeting ID may serve as a useful additional treatment strategy for patients with chronic HF in high-income countries. However, there is limited information on the diagnosis of and therapy for ID in HF in SSA, where infections and malnutrition are more likely to influence the situation. This article reviews the present epidemiological gap in knowledge about anaemia and ID in HF, as well as the diagnostic and therapeutic challenges in SSA.

## Introduction

The importance of anaemia in heart failure (HF) has attracted considerable interest over the past two decades. Recently, iron deficiency (ID) with or without anaemia has been recognised as an emerging therapeutic target with prognostic implications.^1^-^5^ In both conditions, the epidemiology, diagnosis and therapies have been extensively studied in developed countries but are largely unexplored in sub-Saharan Africa (SSA), where infections and malnutrition are common and may influence the situation.^1^

This article focuses on the present epidemiological gap in knowledge about anaemia and ID in HF, as well as the diagnostic and therapeutic challenges in SSA. No formal search of Medline or other search engines was performed; however, PubMed and Cochrane were checked for all relevant articles. The criterion to include an article was clinical relevance. Full versions of articles rather than abstracts were assessed for inclusion.

## Defining anaemia in the setting of HF in SSA and its clinical relevance

The definition of anaemia has a definite impact on the burden of anaemia in patients with HF, which also varies according to the setting and population in which anaemia is being considered. The precise cut-off values to define anaemia in HF are arbitrary and there is no consensus as to the definition of anaemia specific to patients with chronic diseases such as HF.

The historical cut-off points put forward by the World Health Organisation (WHO), namely a haemoglobin (Hb) concentration < 13 g/dl for men or < 12 g/dl for women have been under debate regarding their relevance when it comes to SSA, where haemoglobin values have been reported to be relatively low in the normal general population.^6^,^7^ This difference might be related to a high prevalence of infections, haemoglobinopathies and nutritional deficiencies. Furthermore, genetic factors may also be implicated.^6^,^7^ In SSA, the concept of clinically relevant Hb cut-off points has been applied in some settings, leading to the use of more stringent cut-offs when reporting anaemia in HF in SSA compared to studies from high-income countries.^2^,^8^

For example, a Hb cut-off value of < 10 g/dl in HF for both genders was used in the Tanzania Heart Failure (TaHeF) study,^2^ the SSA Survey of Heart Failure (THESUS) study,^8^ and by Ogah et al.,9 while in the Heart of Soweto,^10^ a cut-off value of < 11 g/dl for men and < 10 g/dl for women was defined as clinically relevant anaemia (Table 1). This further complicates the comparability and potential criteria for interventions versus what has already been reported from high-income countries. Accordingly, there is a need for standardised and uniform cut-off points that are relevant to and applicable in SSA.

## Epidemiological gap in knowledge of anaemia burden in HF in SSA

The available data suggest that there are limited reports about the epidemiology of anaemia in SSA compared to a large number of studies in high-income countries. Using the WHO cut-off point, the small amount of scattered information available reveals that the prevalence of anaemia in HF in SSA ranges from 14 to 64% (45% on average) (Table 1), compared to 36% in the general population. In high-income countries, the prevalence ranges from 10 to 49% (34% on average), compared to 8% in the general population.^11^,^12^

**Table 1:** Studies in SSA reporting on adult HF patients with anaemia

*Authors, country and year*	*Sample size*	*Anaemia (%)*	*Definition of anaemia by haemoglobin (g/dl) or packed cell volume (%)*
Makubi et al.^2^ Tanzania, 2015			
Ogah et al.^9^ Nigeria, 2014	452	8.8	< 10
Damasceno et al.^8^ 9 African countries, 2012	1006	15.2	< 10
Stewart et al.^10^ South Africa, 2008.	699	10.0	Male < 11, female < 10
Karaye et al.^13^ Nigeria, 2008	79	41	< 39% in male and < 36% in female
Kuule et al.^14^ Uganda, 2009	157	64.3	Male ≤ 12.9, female ≤ 11.9
Inglis et al.^15^ South Africa, 2007	163	13.5	World Health Organisation
Kuule et al.^16^ Uganda, 2009	140	15.7	Not available
Oyoo et al.^17^ Kenya, 1999	91	13.2	Not available
Ojji et al.^18^ Nigeria, 2013	475	8.0	Not available
Onwuchekwa et al.^19^ Nigeria, 2009	423	6.2	Not available

Higher rates of prevalence are therefore seen in SSA than in high-income countries, and in both populations, the prevalence of anaemia in HF is higher than the global burden of anaemia in the general population. Less attention is paid by clinicians in SSA to screening for anaemia in HF in a clinical perspective, which may be explained by the scarcity of epidemiological data.

As far as interventions are concerned, there are no clinical trials in SSA that provide guidance on the appropriate approach to manage anaemia in HF. Due to the relatively recent attention given to the importance of iron deficiency in HF in SSA, guidelines do not provide help in this regard. Studies are therefore needed to provide more insight into the burden, peculiarities and possible interventions for anaemia in HF in SSA.

## Epidemiological gap in knowledge of ID burden in HF

The prevalence of ID in HF populations in SSA is largely unknown. To our knowledge the TaHeF study, reporting a prevalence of 67%, was the only study providing data on the prevalence of ID in HF in SSA.^2^This should be seen in the perspective of more than 12 studies from high-income countries (Table 2). Since the only study so far conducted indicates that iron-deficiency anaemia is a very common condition in SSA, further studies should aim to see whether active detection and correction of ID are warranted.

## Challenges in biochemical diagnosis of ID in HF

Absolute ID is conventionally defined by a serum ferritin level of < 30 mg/l.^29^,^30^ As the ferritin is elevated in HF due to the inflammatory state, in their 2012 guidelines, the European Society of Cardiology introduced the definition of ID in HF as either serum ferritin < 100 mg/l for absolute ID or 100–299 mg/l and transferrin saturation < 20% for functional ID.31 The criteria have been used in several clinical trials.^32^-^34^ These diagnostic criteria for ID in HF used in high-income countries may not be feasible in SSA due to the lack of diagnostic facilities and the presence of co-existing malnutrition, haemoglobinopathies and infections.

Serum ferritin/transferrin saturation (TSAT) has commonly been used in several observational and clinical trials (Table 2) to diagnose ID in HF in high-income countries. The unavailability of biochemical iron markers in many SSA countries may limit the use of these diagnostic criteria as applied in high-income countries and this may underestimate the magnitude of iron deficiency in this population.

**Table 2:** Studies reporting on the magnitude of ID in HF

*Authors, country and year*	*Number*	*% with ID*	*Definition of ID*
Makubi et al.^2^ Tanzania, 2014	411	67	MCV < 80 fl
Jankowska et al.^20^ Poland, 2014	165	37	Low hepcidin and high sTfR Serum ferritin and TSAT
Rangel et al.^3^ Portugal, 2014	127	36	SF < 100 μg/l OR SF 100–299 μg/l +TSAT < 20%
Parikh et al.^21^ United States, 2014	574	61	SF < 100 μg/l OR SF 100–299 μg/l +TSAT < 20%
Enjuanes et al.^22^ Europe, 2014	1278	58	SF < 100 μg/l OR SF 100–299 μg/l +TSAT < 20%
Ijsbrand et al.^4^ Europe, 2014	1506	50	SF < 100 μg/l OR SF 100–299 μg/l +TSAT < 20%
Jankowska et al.^23^ Poland, 2013	443	35	SF < 100 μg/l OR SF 100–300 μg/l +TSAT < 20%
Nanas et al.^24^ Greece, 2006	37	73	Bone marrow
Cohen-Solal et al.^25^ France, 2014	832	72	SF < 100 μg/l OR SF 100–299 μg/l +TSAT < 20%
Yeo et al.^26^ Singapore, 2014	751	61	SF < 100 μg/l OR SF 100–299 μg/l +TSAT < 20%
De Silva et al.^27^ UK, 2006	955	29	Lower limit for serum iron and SF
Klaus et al.^28^ UK, 2004	296	14	Low SF

sTfR: soluble transferrin receptor, TSAT: transferrin saturation, SF: serum ferritin, TR: transferrin receptor.

Red cell indices such as mean corpuscular volume and the degree of hypochromia, which are used in many SSA countries, cannot distinguish between the presence or absence of sufficient bone marrow iron in patients with chronic disease, thereby offering a relatively low sensitivity (Table 3).35 This information gap warrants serious attention if ID is to be intervened in by the provision of diagnostic resources, allowing the use of serum ferritin, which provides a considerably higher specificity and sensitivity compared to haematological indices (Table 3).

**Table 3:** Sensitivity and specificity of iron measures in chronic diseases

*Study, year*	*Iron marker*	*Sensitivity (%)*	*Specificity (%)*
Punnonen et al.^36^ 1996	% hypochromia	77	90
Punnonen et al.^36^ 1996	Mean corpuscular volume	86	
Means et al.^37^ 1999		42	83
Punnonen et al.^36^ 1996	% transferrin saturation	79	
Means et al.^37^ 1999		38	89
Van Tellingen et al.^38^ 2001	Serum ferritin	79	97
Lee et al.^39^ 2001		87	
Punnonen et al.^36^ 1996		89	
Joosten et al.^40^ 2001		94	95

## Absolute ID and serum ferritin < 60–100 μg/l in HF

It has been suggested that cut-off levels of the order of 60–100 μg/l of ferritin rather than the normal < 30 μg/l, or indeed previously reported 12–15 μg/l, should be used when screening for absolute ID in people with co-existing inflammation, infection and malignant conditions.^29^,^30^,^41^ This recommendation is based on the fact that patients with acute or chronic disease usually have elevated ferritin levels as a result of intracellular iron accumulation and the inflammatory response. The explanation is that serum ferritin is an acute-phase reactant. Even these higher levels only slightly improve the sensitivity (Table 3).

The combined use of serum ferritin with inflammatory markers such as erythrocyte sedimentation rate (ESR) or C-reactive protein (CRP) in a discriminant analysis provide only marginal improvement in sensitivity/specificity.42 Serum ferritin < 100 μg/l has been widely used as a cut-off in high-income countries when looking for absolute ID in patients with HF in most clinical trials. Studies supporting its use in SSA are limited.^34^,^43^,^44^

Serum ferritin levels such as < 150 μg/l offer a better balance between sensitivity and specificity than < 100 μg/l (Table 4).29,39 Afro-Americans and black Africans tend to have a high level of serum ferritin.^45^,^46^ It is not clear whether this is genetic or due to environmental changes as a result of common chronic infection. In view of this, high cut-off values such as < 150 μg/l (rather than < 100 μg/l) may be more appropriate but this requires further study and validation. Such studies will pave the way to clinical trials of relevance to SSA.

**Table 4:** Sensitivity and specificity of serum ferritin

*Author, year*	*Ferritin cut-off value (ng/ml)*	*Sensitivity (%)*	*Specificity (%)*
Lockhat et al.47 2004	< 50	37	75
	< 100	48	75
	< 150	71	69
	< 200	77	37
Tessitore et al.^48^ 2001	< 100	35	78
Kalantar-Zadeh et al.^49^ 2004	< 200	41	100

## Treatment approaches with regard to iron therapy in HF

## Utility and beneficial effect of iron therapy in HF

In a series of controlled and uncontrolled clinical trials of Table 3. Sensitivity and specificity of iron measures in chronic diseases HF and ID (Table 5), all conducted in high-income countries, parental iron showed clear short- to medium-term benefits, leading to improved symptoms and quality-of-life measures and less hospitalisation.^32^,^33^,^50^-^53^ In the FAIR-HF study, patients were randomised to parental iron or placebo and 50 versus 28%, and 47 versus 30% reported improved quality of life and New York Heart Association (NYHA) class, respectively.^32^ Similarly in the FERRIC-HF study, 35 patients with congestive heart failure were put on 16 weeks of intravenous iron or no treatment in a 2:1 ratio.^50^ The NYHA functional class improved in eight patients (44%) in the iron group versus no patients in the control group (p = 0.03).

In all these trials, parental iron was used as a supplement, added to standard therapy on optimal pharmacological treatment, which included a diuretic, a beta-blocker and/or an angiotensin converting enzyme (ACE) inhibitor or angiotensin receptor blocker (ARB) as determined by the investigator (unless contra-indicated or not tolerated). Data on the efficacy of parental iron remain undisclosed in SSA.

**Table 5:** Studies on parental iron therapy in HF

*Author, year*	*Study design*	*Sample size*	*Type of parental iron*	*Dose/duration*	*Benefits*
Ben-Assa et al.^54^ 2015	Uncontrolled	34	Ferric sucrose	200 mg, 6 weeks	↑Hb
Reed et al.^53^ 2015	Uncontrolled	13	Ferric gluconate	250 mg bd/day, 3 days	↑Hb, ↑SF, ↑TSAT
Gaber et al.^55^ 2011	Uncontrolled	40	Ferric dextran	200 mg/week, 4–8 weeks	NYHA, ↑6MWD, ↑SF, ↑TSAT, ↑exercise capacity, ↑renal function, ↑QoL
Usmanov et al.^52^ 2008	Uncontrolled	32	Ferric sucrose	100 mg 3×/week, then once/week, 26 weeks	↑Hb, ↑NYHA, ↑LV diameters
Bolger et al.^56^ 2006	Uncontrolled	16	Ferric sucrose	1 g daily, 12 days	Hb 12.55, ↑TSAT, ↑6MWD ↑NYHA
Toblli et al.^57^ 2015	60	Ferric sucrose	200 mg/week, 5 weeks	↑Hb, ↑SF, ↑TSAT, ↑LV diameters, ↑LVEF, ↑ CrCl, ↑NT-proBNP	Ponikowski et al.33 2014
Ponikowski et al.33 2014	Controlled	304	Ferric carboxymaltose	Total dose 500–2000 mg, in correction phase 500 mg, in maintenance 52 weeks	↑6MWD, ↑NYHA, ↑exercise capacity, ↑PGA, ↑QoL, ↑ hospitalisation, ↑fatigue score
Terrovitis et al.^58^ 2012	Controlled	40	Ferric sucrose	300 mg weekly, 6 weeks	↑Hb
Anker et al.^32^ 2009	Controlled	459	Ferric carboxymaltose	200 mg, 24 weeks	↑Hb, ↑SF, ↑TSAT, ↑PGA, ↑NYHA, ↑6MWD, trend ↓hospitalisation
Drakos et al.^59^ 2009	Controlled	16	Ferric sucrose	300 mg/week, 6 weeks	↑Hb
Arutyunov et al.^60^ 2009	Controlled	30	Ferric carboxymaltose Ferric sucrose	200 mg weekly to calculated dose, then 200 mg every 4 weeks, 12 weeks	Not applicable
Okonko et al.^50^ 2008	Controlled	35	Ferric sucrose	200 mg weekly, 16 weeks	↑Hb, ↑SF, ↑VO2, ↑exercise capacity, ↑NYHA, ↑PGA
Toblli et al.^61^ 2007	Controlled	40	Ferric sucrose	200 mg/week, 5 weeks	↑Hb, ↑NT-proBNP, ↑LVEF, ↑NYHA, ↑exercise capacity, ↑renal function: ↑QoL

Hb: haemoglobin, SF: serum ferritin, TSAT: transferrin saturation, NYHA: New York Heart Association, 6MWD: six-minute walking distance, QoL: quality of life, LV: left ventricular, LVEF: left ventricular ejection fraction, NT-proBNP: N-terminal pro B-type natriuretic peptide, CrCl: creatine clearance rate, PGA: patient’s global assessment, pVO2: peak oxygen consumption, ↑: improved.

## Dosage for parental iron therapy in HF

Table 5 provides the dosage for various types of parental iron used in clinical trials, nine of which used parental ferric sucrose (FSC),^24^,^52^,^54^,^56^,^57^,^61^-^63^ two used parental ferric carboxymaltose (FCM),^32^,^33^ one study used both ferrics,^60^ one used ferric gluconate,^53^ and one iron dextran.^55^ In most of the studies, the 200-mg weekly dose for parental FSC was applied in the correction phase, with a maintenance period in some studies. However, for parental FCM, it was given either as a total loading dose to correction or a 200-mg weekly dose. There is therefore a need to have a standardised dose for both parental FSC and FCM, and to determine whether the same doses apply in SSA.

## Treatment targets of parental iron therapy in HF

The target treatment levels are variable, ranging from replenishment through maintenance to a predetermined period of study or haemoglobin level. From the clinical perspective, this needs to be carefully determined from additional studies, for guideline implementation. The levels of haemoglobin for initiation and cessation should also be properly studied, as well as the period of maintenance or monitoring for those who receive iron replenishment.

## Long-term effects after parental iron therapy in HF

During treatment, intravenous iron seems to be relatively safe with only a few side effects or adverse events, which can usually be tolerated by the patients.^33^,^44^ However, data are limited on the long-term effects after this therapy is ended, such as undesirable complications (iron overload or myocardial changes) several years after therapy. It is also not known how long the replenished iron store and improved clinical symptoms of HF are sustained following parental iron therapy. A close follow up of patients who received iron therapy, several months or years after therapy may shed some light on the matter.

## Excluded populations in parental iron therapy trials

Despite the significant progress made in the use of parental iron in patients with HF and ID, most of the trials included patients with heart failure with reduced ejection fraction (HFrEF) (EF < 40 or 45%) and no data are available for patients with heart failure with preserved ejection fraction (HFpEF). It is also unclear whether this therapy could benefit patients with HF due to valvular heart disease, obstructive cardiomyopathy, those with Hb levels < 9.5g/dl or > 13.5g/dl and iron deficiency. The findings from these trials therefore cannot be generalised and must be applied with caution in SSA populations.

## Possible limitation of parental iron therapy in SSA

The high level of iron deficiency in a setting where infections, haemoglobinopathies and malnutrition are common requires special attention.^2^ The role of parental iron therapy (and other potential options) in SSA requires further justification before implementation measures are considered. The TaHeF study, along with a few other reports from SSA, have locally quantified the magnitude of anaemia, as shown in Table 2.^2^

TaHeF was the only study that characterised ID, which resulted in a poor prognosis in HF patients. With this limited regional data, further studies are needed to identify the peculiarities of ID and other types of anaemia or nutritional deficiencies (folate, vitamin B_12_) in HF in SSA and determine whether the consequences are the same as in high-income countries before any interventions (whether parental or oral) are conducted or adopted.

Apart from epidemiological challenges, as explained above, the other important limitation may be related to acceptance of and adherence to parental iron. Across all studies done in high-income countries, none looked at the level of adherence. Even with oral therapy and other HF medication, the problem of compliance in SSA is high and is mainly related to financial constraints, limited access to health facilities, as well as limited health education/awareness. Proper measures should therefore be put in place to address this.

This approach also imposes a burden on the patient, with increased clinic appointments and transportation costs, and absence from work of people with already reduced mobility and functional capacity. This may complicate the already compromised health system with overloading of clinics and administrative logistics. There is possibly a need to have an accelerated iron-supplementation regimen, which would shorten the duration, or look into the possibility of providing parental iron for replenishment in the hospital ward, while maintenance with oral iron is taken at home, with more widely scheduled appointments.53 Finally, parental iron is expensive and administration to large populations of HF patients may not be feasible, particularly in countries with limited healthcare resources.

## Possible role of oral iron therapy in SSA

Oral iron supplementation is an established therapy for treating iron deficiency in a range of medical conditions but it has not been widely tested in HF patients. It remains promising in resource-limited settings because (1) newer ferrous sulphate preparations may be better absorbed than the older ferrous sucrose; (2) the pathophysiology or iron deficiency may differ geographically; and (3) oral iron supplementation is inexpensive.

Preliminary studies (Table 6) on randomised clinical trials on erythropoiesis-stimulating agents (ESA) versus oral iron supplementation showed no improvement in exercise capacity or Hb and ferritin levels with oral therapy. However in a recent non-randomised clinical trial,^64^ the researchers found that replenishment of Hb, TSAT and ferritin produced similar results to giving parental iron in the FAIR trial^32^ in patients with HF. A randomised trial^62^ also showed ferritin and Hb levels increased when using both parental and oral iron, although the study was underpowered.

**Table 6:** Study reporting oral iron therapy as an interventional drug or placebo in HF

*Author, year*	*Study design*	*Sample size*	*Type of iv iron*	*Dose*	*Target dose*	*Benefits*	*Adverse effect/toxicity*
Niehau et al.^64^ 2015	Observational	105	Oral iron (NS)	NS, 180 days	Iron repletion	↑Hb, ↑SF, ↑TSAT, ↑Iron, ↑TIBC	NR
Tay et al.^65^ 2010	Observational	Observational	Ferrous fumarate	200 mg 3×/day, 12 weeks	Iron repletion	Hb, ferritin, TSAT, 6MWT	No adverse effect
Beck-da-Silva et al.^62^ 2013	Controlled	18	Ferrous sulphate	200 mg 3×/day, 8 weeks	NR	↑Hb, ↑Ferritin, ↑TSAT, ↑peak VO2, ↑NHYA	NR
Parissis et al.^66^ 2008	Controlled	24	Ferrous sulphate	250 mg twice a day, 12 weeks	NR	No change in QoL, Hb, significant deterioration in exercise capacity	1 TIA, 1 constipation
Van Velduisen et al.^63^ 2007	Controlled	165	Oral iron	200 mg/day, 26 weeks	NR	No change in exercise capacity, Hb, ferritin, TSAT, minor improvement in QoL, NYHA class	Adverse effect comparable to ESA including discontinuation, HF, HT, DVT
Palazzuoli et al.^67^ 2006	Controlled	40	Ferrous gluconate	300 mg/day 12 weeks	NR	No changes in NYHA, exercise capacity, Hb, BNP, creatinine	NR

iv: intravenous, NS: not specified, NR: not reported, Hb: haemoglobin, SF: serum ferritin, TSAT: transferrin saturation, NYHA: New York Heart Association, 6MWD: six-minute walking distance, VO2: oxygen consumption, QoL: quality of life, TIBC: total iron-binding capacity, TIA: transient ischaemic attack, ESA: erythropoiesis stimulating agent, DVT: deep-vein thrombosis, HT: hypertension, BNP: B-type natriuretic peptide.

**Table 7:** Key points

• Anaemia and ID are both common in HF and have prognostic implications
• Although intravenous iron supplementation appears to be beneficial in the treatment of patients with HF and ID, oral iron supplementation may be a potential alternative in resource-limited countries such as in SSA.
• Studies are needed to provide more insight into the burden and peculiarities of and intervention for anaemia and ID in HF in SSA, in which the pathophysiology may be different from that in high-income countries.
• In both conditions, the epidemiology, diagnosis and therapies have been extensively studied in developed countries but are largely unexplored in SSA

In a prospective study of 25 patients with cyanotic congenital heart disease, the researchers demonstrated a significant improvement in serum ferritin and Hb levels and the six-minute walking test (6MWT) distance after 90 days of oral iron supplementation with 200 mg iron fumarate three times per day.^65^ The recently completed TaHeF-ID study has also shown similar findings, with additional improvement in left ventricular ejection fraction from 37.8 ± 12.2% to 44.5 ± 10.7% (+17%; p < 0.001) and N-terminal pro B-type natriuretic peptide (NT-proBNP) from 986 ± 774 ng/l to 582 ± 503 ng/l (–41%; p < 0.001) from baseline after 90 days of a similar dosage of iron sulphate.^68^

These findings are promising and justify randomised clinical trials to address this area of uncertainty by comparing parental and oral iron supplementation, particularly in SSA. Results from the IRONOUT trial (NCT02188784), which is being conducted by the National Heart, Lung, and Blood Institute’s Heart Failure Network are also awaited.^69^

## Conclusions

The accumulating data on HF and anaemia/ID anaemia continue to be largely of studies conducted in high-income countries, with very limited information for SSA. Creating awareness and identification of these co-morbidities in HF, both in the hospital setting and at the population level, should be a priority. Diagnostic dilemmas and therapeutic challenges require further exploration in SSA, in which the pathophysiology of ID in HF and the healthcare system may differ from that of high-income countries.
